# On the Sorbent Ability and Reusability of Graphene-Oxide–Chitosan Aerogels for the Removal of Dyes from Wastewater

**DOI:** 10.3390/gels9020110

**Published:** 2023-01-27

**Authors:** Filippo Pinelli, Chiara Piras, Liebert Parreiras Nogueira, Filippo Rossi

**Affiliations:** 1 Department of Chemistry, Material and Chemical Engineering “Giulio Natta”, Politecnico di Milano, via Mancinelli, 7, 20131 Milan, Italy; 2 Oral Research Laboratoy, Institute for Clinical Dentistry, University of Oslo, NO-0317 Oslo, Norway

**Keywords:** aerogels, chitosan, graphene oxide, regeneration, wastewater treatment

## Abstract

One of the most persistent issues affecting people worldwide is water contamination due to the indiscriminate disposal of pollutants, causing severe environmental problems. Dyes are among the most harmful contaminants because of their high chemical stability and consequently difficult degradation. To remove contaminants from water, adsorption is the most widely used and effective method. In this work, we recall the results already published about the synthesis, the characterization and the use of porous graphene-oxide–chitosan aerogels as a sorbent material. Those systems, prepared by mixing GO sheets and CS chains, using APS as a cross-linking agent, and by further lyophilization, were further characterized using nano-computed tomography, supplying more understanding about their micro and nano-structure. Their sorbent ability has been investigated also by the study of their isotherm of adsorption of two different anionic dyes: Indigo Carmine and Cibacron Brilliant Yellow. Those analyses confirmed the potentialities of the aerogels and their affinity for those anionic dyes. Moreover, the possibility of regenerating and reusing the material was evaluated as a key aspect for applications of this kind. The treatment with NaOH, to promote the desorption of adsorbed dyes, and subsequent washing with HCl, to re-protonate the system, ensured the regeneration of the gels and their use in multiple cycles of adsorption with the selected water contaminants.

## 1. Introduction

Aerogels are generally defined as crosslinked materials characterized by softness and exceptional porosity obtained through freeze drying from a physical gel precursor [1]. They are stretchable, large in specific area, have great thermal and electrical properties and have the ability to detect and react to external stimuli [2,3]. Depending on the nature of their components, aerogels can be biocompatible and have applications in the biomedical field and as sensors, adsorbent materials and catalyst supports [4,5,6,7]. Moreover, in the last years, many researchers have focused on graphene and graphene-oxide-based systems thanks to the features and characteristics of these molecules. Specifically, graphene oxide, an oxidized form of graphene made of a single monomolecular layer with various oxygen functional groups, presents great elasticity and flexibility, resistance to mechanical stimuli, adsorbent features and unique surface chemistry, making this molecule an ideal candidate for aerogels synthesis [8,9]. Due to this, many different systems of this kind have been designed in recent years for environmental applications and for wastewater treatment, and valuable strategies are already available in literature [10,11,12,13,14,15]. A notable example is represented by the synthesis of doped reduced-graphene-oxide aerogels developed by controlled self-assembly tactics and applied to remove radioactive cesium from effluent [16]. The three-dimensional layered structure ensures for the system excellent mechanical strength and a large specific surface area. It also facilitates exposure of adsorption sites, which promotes the adsorption of contaminant ions—in this case, Cs^+^. The composite materials exhibited excellent adsorption performances and Cs+ selectivity: the maximum adsorption capacity was 227 mg/g, and the removal rate was more than 90%. Another example of a functionalized aerogel for efficient and selective removal of ions is represented by graphene oxide (GO)–montmorillonite (MMT) composite systems [17]. This framework has controllable slit-shaped pores, and thanks to the combination of MMT with GO, great selectivity for hydrated copper ions in wastewater systems is ensured. Great removal efficiency, excellent selectivity and regeneration for up to eight cycles confirmed the effectiveness of these devices.

Similarly, in 2021, Shadkam et al. proposed the synthesis and applications of reduced-graphene-oxide–cellulose-nanocrystal hybrid aerogels [18]. The combination of those reagents ensured reinforcement in the framework of the system, which is always a desired feature for devices of this kind and applications, and obviously, excellent adsorbent capacity was reported. Moreover, the adsorption behavior of the material was described by Langmuir isotherms with a maximum adsorption capacity of 454 mg/g for the removal of toluene from aqueous media, which is an excellent performance for this kind of application.

The quick overview above shows the great variety of graphene-oxide-based systems available in the literature and their versatility in the adsorption of contaminants. In this context, a very interesting strategy for synthetizing effective composite materials for wastewater treatment is represented by the combination of graphene oxide and chitosan (CS) chains [19,20]. This strategy is very interesting, since it merges the excellent properties of chitosan with those of GO. The final systems have great mechanical resistance, excellent adsorbent properties thanks to their porosity and a combination of the negative charges of GO and the positive charges of CS. A clear example of such a system was presented by Shi et al. They proposed the synthesis of composite aerogels of these two molecules, prepared with a 1–2 ratio between GO and CS [21]. The system showed efficient adsorption ability with methyl orange (MO) and methylene blue (MB) from water and pH responsive behavior for single-dye adsorption, tending to adsorb MO at a low pH and MB at a high pH because of hydrophobic adsorption and electrostatic interactions between the framework and the external environment. Similarly, systems of this kind can also be designed as microdevices. A notable example of this is represented by composite aerogel microspheres of chitosan and graphene oxide fabricated via CO_2_ supercritical drying, which displayed excellent performance for bilirubin removal [22]. Those systems showed good mechanical resistance, great surface area (175 m^2^/g) and a pore-size distribution of 20–40 nm. Large adsorption capacity was observed (178 mg/g within 2 h), and the Freundlich model, ascribed to multilayer adsorption, fitted well the adsorption isotherm.

As presented above, various systems based on graphene oxide are employed in wastewater treatment, and their combination with chitosan ensures effective devices. In this work, we propose for the first time the realization of GO-CS aerogels with controlled porosity and the use of ammonium persulfate (APS) as a crosslinking agent, depending on dyes’ adsorption and regeneration. Moreover, the adsorption isotherm for the adsorbent was investigated, together with the possibility of regenerating it in an efficient, solvent free and not-expensive way to reduce the adsorbent required and have a lower environmental impact [23,24,25].

## 2. Results and Discussion

### 2.1. Aerogel Network Formation

**Figure 1:**
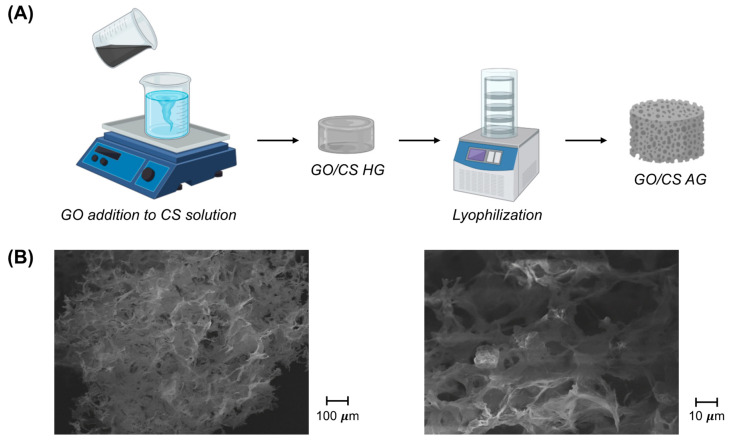
(**A**) Schematization of the synthetic procedure for GO/CS AG formation. (**B**) SEM images at two different magnifications, as indicated.

The network of the synthetized material was obtained through the cross-linking between graphene oxide and chitosan chains. Graphene oxide can be dispersed in water because of the electrostatic repulsion between their sheets, and the presence of chitosan in the solution can lead to a physical change in the system [26,27,28]. Chitosan is a positively-charged polymer that is able to balance the negative charges of graphene and strongly attract its molecules. These electrostatic interactions determine the hydrogen bonding and the formation of the precursor hydrogels. The chemical characteristics of the system were studied by working with ATR-FTIR and are presented in the Supplementary Materials [23], where the key features of the molecules of chitosan and graphene oxide can be detected in the spectra of GO-CS aerogels with few modifications in parameters, such as intensity or a shift towards a higher wavenumber, due to the linkage between the polymer and the graphene oxide [29]. In Figure 1, we report the schematization of the synthetic procedure together with the SEM analysis; additional SEM analysis is available in Figure S2 (Supplementary materials). From SEM images, it is possible to observe the 3D porous structure with pore sizes in the order of microns together with its uniformity, underlining the efficient mixing procedure used.

**Figure 2:**
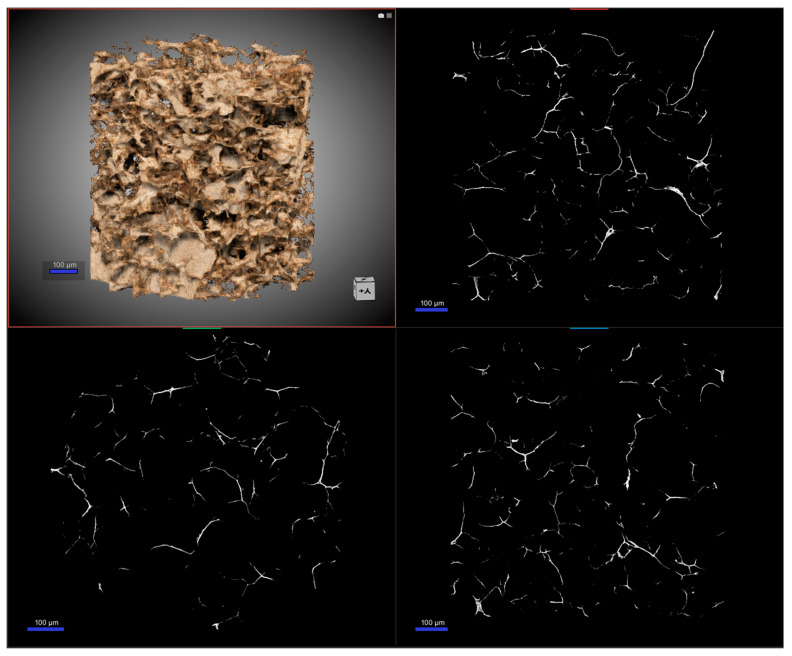
Nano-CT images of the GO-CS aerogel. On the top left, the 3D reconstruction of the gel is reported, and in the other panels, the reconstruction, as a 2D map, of the internal porosity paths from different angles is visible.

Here we continue the discussion by introducing the nano-computed tomography (nano-CT) as an effective tool to get additional information on the framework of the gel. Nano-CT is an innovative high-resolution cross-sectional imaging technique using X-rays to create cross-sections starting from a construction object. We built a virtual model of the specimen with the advantage of not destroying the original sample [30,31]. Based on the application of a transmission-target X-ray tube, the focal spot size can be reduced to diameters less than 400 nm. Thanks to specific detectors and examination protocols, a superior spatial resolution of up to 400 nm can be achieved, exceeding the resolution capacity of typical micro-CT systems. In Figure 2, the results of nano-CT for the graphene-oxide–chitosan aerogels are reported.

The structure appeared homogeneous, and the analysis demonstrated a highly porous system, almost like a foam. The pores were in the scale of 25–75 µm, with irregular shape and no directional bias on them, as shown in the 2D map. The total porosity was found to be more than 96%, and almost all of it was classified as open porosity—interconnected pores; the structure thickness was estimated to be in the order of 3 µm. Moreover, we considered the final application of the system to be as an adsorbent material, so another important parameter is the pore connectivity, which was quantified as a function of the minimum pore diameter considered. This led to a connectivity density of the total pore space of 3 × 10^−5^ µm^−3^.

Lastly, a parameter that is configured more in the imaging field is the structure separation. In fact, it represents the thickness of the space by the binarization within the image. Values of this factor can be calculated from 2D images. In this case, we obtained 72 µm with structure linear density of 0.01 µm^−1^. The results obtained with nano-CT confirmed and expanded the outcomes obtained with scanning electron microscopy already discussed. The porous nature of the framework of the aerogel was verified, along with the homogenous distribution of the pores. Moreover, the additional details presented regarding the features of the system, the pore distribution and their connectivity, corroborated the use of the materials for the selected application [32]. In Table S1, in the Supplementary Materials, a complete dataset of the parameters obtained from the nano-CT is reported.

### 2.2. Adsorption Tests and Isotherm Study

**Figure 3:**
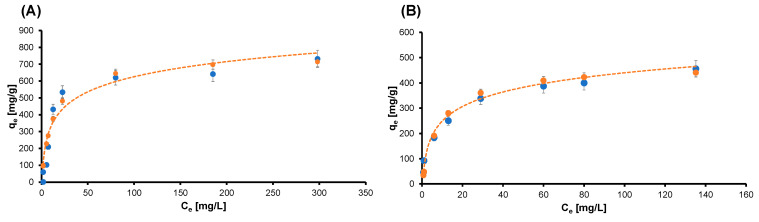
Plot of the collected data and experimental model for the isotherm curve of (**A**) CBY and (**B**) IC. Blue dots are the experimental data, and the orange curve is the Langmuir model. Data are presented as means ± standard deviations.

The adsorption kinetic tests were conducted as described in the previous section, with the solutions with different concentrations. The collected data were plotted as time-dependent adsorption curves—sorption capacity (q) versus time—and as percentage of dye removed versus time. In all the considered cases, fast kinetics of adsorption were observed, and the efficacy of those systems was confirmed. The obtained results are consistent with the ones already published, and therefore, we are not reporting them here again. Next, we can deepen the discussion about the adsorption ability of the graphene-oxide–chitosan aerogels by investigating the isotherm of the adsorption of the system. The tests were conducted as described in the Materials and Methods section, and we modeled the data using the Langmuir equation. The Langmuir adsorption isotherm can model the equilibrium between an adsorbate and an adsorbent system, where the adsorption is limited to one molecular layer, while considering the surface as homogeneous and assuming that there is no lateral interaction between adjacent adsorbed molecules when a single molecule occupies a single surface site [33,34]. Quite accurate modeling was obtained using the Langmuir equation, and the results are reported in the Figure 3.

In both cases, the dataset of the dyes was fitted with good accuracy by the model (coefficient of determination higher than 0.9), suggesting that monolayers of CBY and IC dye molecules were adsorbed uniformly on the sorption site. Moreover, the steep slope of the first portion of each curve, i.e., the high sorption capacity (q_e_) with a low concentration of the pollutant, confirmed the results of the kinetic sorption test. The maximum value of the adsorption capacity Q_max_ and the value of the Langmuir constant K are reported in Table 1.

From the data presented above, it can be observed that the value of the Langmuir constant of the model obtained for the IC (K = 0.11 L/mg) is higher than the one resulting from the curve of the CBY (K = 0.07 L/g), highlighting a higher sorbent-solute affinity for IC than CBY. Regarding saturation, the dyes reached approximately the same value in moles, and this could be explained by their similar hydrodynamic radii [35]. In Table 2, we report a comparison among the adsorption capacities of various adsorbent materials described in the literature which are employed in the adsorption of IC and CBY. The GO/CS aerogel is for certain among the most interesting materials for this application.

### 2.3. Desorption and Reusability Test

**Figure 4:**
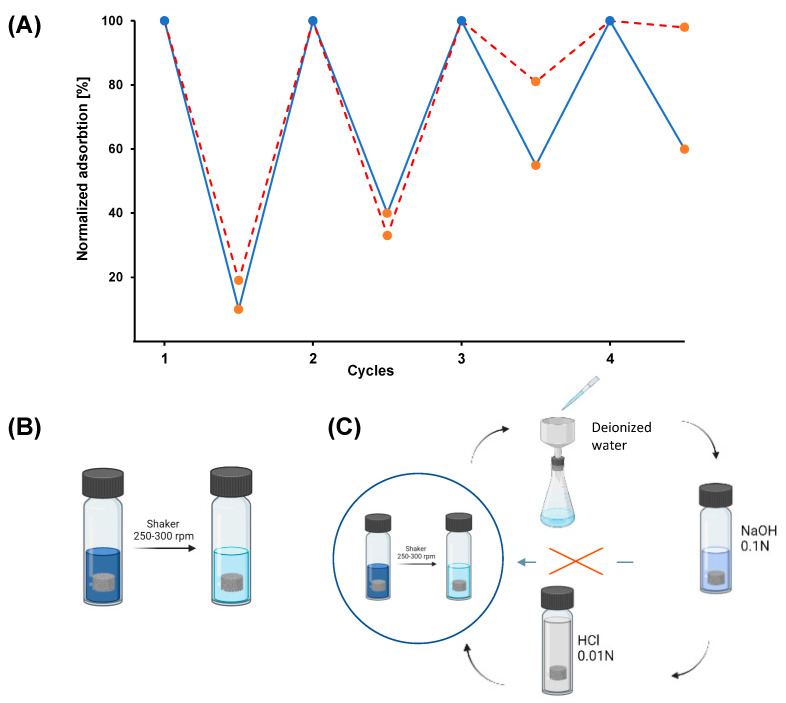
(**A**) Normalized adsorption (%) of the material versus the cycle of adsorption. The blue line represents the Indigo Carmine profile, and the dashed red line represents the Cibacron Brilliant Yellow profile. Blue dots represent the starting point for the adsorption test of each adsorption cycle, and orange dots represent the end of the adsorption cycle. (**B**) Schematization of the adsorption procedure. (**C**) Schematization of the regeneration procedure.

To further study the potential of GO/CS aerogels as sorbent materials, the possibility of regenerating and re-using the system after initial sorption was evaluated. The reusability of the material is a pivotal aspect in applications of this kind, since it reduces the need for new adsorbent and the problem of disposal of the used one, reducing the overall environmental impact [41]. The reusability of GO/CS aerogels was assessed by conducting four cycles of adsorption–desorption in which lower volumes of de-sorbent solution were used with respect to the treated solution (approximately 4–5 times). As described in the previous section, after each cycle of adsorption, the used samples were treated with NaOH 0.1 N to promote the desorption of the dyes. In fact, given the presence of sulphonate groups, they can be easily dissolved in an alkaline environment, promoting their desorption from the aerogel framework [42]. This treatment induces a loss of efficacy in the absorbing capacity of the material towards the dyes studied due to the modification of the electrostatic properties of the system induced by NaOH [43]. Due to this, the samples were treated with HCl 0.01 N to re-protonate its structure, regenerating its sorption capacity. After the complete regeneration procedure, the material is ready to be reused. In Figure 4, the results of the regeneration procedure are reported as normalized adsorption (%) versus the cycle of adsorption for the two dyes, together with the schematization of the regeneration and re-use procedure. In both cases, it is possible to see that after the first cycle of adsorption–in which more than 90% of IC was removed and more than 80% of CBY was removed–the sorbent ability of the aerogel decreased such that around 60% of dye was removed in the second cycle of adsorption in both cases. During the third cycle of adsorption, the most important differences between the two dyes appeared: 45% of IC was adsorbed in the third cycle and 40% in the fourth cycle; for CBY, 20% was removed in the third cycle, and very low efficiency was detected during the last cycle.

The decrease in the adsorption capacity, in both cases, has been related to the partial collapse of the internal three-dimensional network of the aerogels and to incomplete desorption and regeneration between the various cycles. This phenomenon was much more evident for CBY; this can be explained by considering that the regeneration procedure is probably more effective with IC due to its higher affinity for the solvent employed for the regeneration. Moreover, some loss of the sample in the form of small fragments was observed during each adsorption and regeneration cycles due to the mechanical and physical stress to which the gel was subjected during the adsorption and regeneration [44]. This occurrence more significant with CBY. This can be explained by considering that the absorption of this dye in the aerogel causes a greater hindrance to the system, which results in a decrease in the mechanical properties of the framework and the subsequent greater loss of material during the tests with the shaker [45].

## 3. Conclusions

In this work, we investigated the use of graphene-oxide–chitosan aerogels—in particular, their adsorbent properties. We confirmed the efficacy of those devices and modeled an aerogel’s isotherm of adsorption for two different dyes using the Langmuir model. This allowed us also to gain more understanding about the affinity of the framework with the employed water contaminants. Moreover, an interesting characterization performed with nano-computed tomography was presented, showing in detail the three-dimensional inner framework of the system and the features of its controlled porosity. Finally, the reusability of the aerogel was demonstrated by performing a desorption and regeneration procedure using NaOH and HCl, without using tensides, guaranteeing multiple cycles of adsorption with the same sample. This work underlined that aerogels are ideal candidates in this kind of application thanks to their high sorbent ability, high surface area, soft nature, responsive behavior, reusability and good mechanical resistance.

## 4. Materials and Methods

### 4.1. Materials

A dispersion of graphene oxide (10 mg/mL) was obtained from GOgraphene, William Blyte Limited (Harlow, Essex, England). Chitosan (at low molecular weight) was bought from Sigma-Aldrich (Sigma-Aldrich Chemie GmbH, Deisenhofem, Germany). All other chemicals were purchased from Sigma-Aldrich (Sigma-Aldrich Chemie GmbH, Deisenhofem, Germany). The materials were used as received.

### 4.2. Synthesis of Graphene-Oxide–Chitosan Composite Aerogels

As explained, aerogels are solid porous materials that, though a sol–gel process, can form a three-dimensional network with high porosity of precursors that are different in nature (inorganic, organic, or hybrid). Here, chitosan–graphene-oxide aerogels were synthetized using an acidic medium, as already described [23]. Briefly, chitosan was dissolved in 2 mL of aqueous acetic acid (2.5% *v*/*v*), and after 2 h the aqueous dispersion of graphene oxide was added. Then, ammonium persulfate, the oxidant agent, was dissolved in water (0.5 mL) and then added to the system to favor the formation of the 3D network. The hydrogels were frozen at −20 °C and then lyophilized to obtain final aerogels. The ratio 1:1.7 between graphene and chitosan was employed.

### 4.3. Characterization of Materials and Nano-Computed Tomography

The aerogels were characterized using scanning electron microscopy (SEM) in order to investigate their inner framework. SEM analyses were obtained using a Zeiss Evo50 with EDS Bruker Quantax 200, and the aerogels were characterized with ATR analysis (attenuated total reflection) using a FT-IR spectrometer from Agilent Technologies, the Varian 640. Spectra were collected under a nitrogen atmosphere at room temperature in the wavenumber range of 400–4000 cm^−1^, with an average of 64 repetitive scans. This was necessary to guarantee a good signal-to-noise ratio together with ahigh reproducibility and a resolution of 4 cm^−1^. Spectra are visible in previous papers [23].

The specimens were then analyzed in a nano-computed tomography device (SkyScan 2211 Multiscale X-ray Nano_CY System, Bruker micro-CT, Kontich, Belgium) using a 20–190 kV tungsten X-ray source and a dual detection system: an 11-megapixel cooled 4032 × 2670 pixel CCD-camera and a 3-megapixel 1920 × 1536 pixel CMOS flat panel.

The samples were scanned at 38 kV, 370 μA and 1400 ms. The scans were taken over 180° with a rotation step of 0.14° and a voxel size of 800 nm using the CCD detector. Projections were reconstructed using the system-provided software, NRecon (version 1.7.4.6), and analyzed with CTAn (Bruker micro-CT, version 1.18.4.0).

pH_PZC_ of aerogels was estimated using methods reported elsewhere [46,47]: the value found was 7.85.

### 4.4. Adsorption Tests

**Figure 5:**
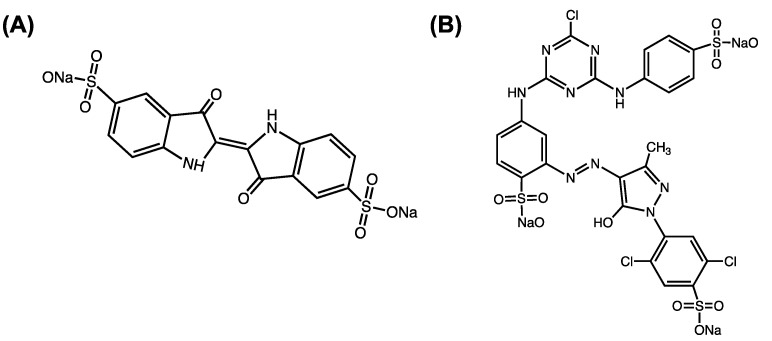
Molecular structures of (**A**) Indigo Carmine and (**B**) Cibacron Cibacron Brilliant Yellow.

The adsorption capacity of the aerogel was studied using an isotherm and kinetic sorption test that was carried out under dynamic conditions, using the shaker at 250–300 rpm at room temperature (25 °C). Indigo Carmine (IC, MW = 466.35 g/mol, λ_max_ = 610 nm) and Cibacron Brilliant Yellow (CBY, MW = 831.02 g/mol, λ_max_ = 402 nm) were employed as contaminants molecules to evaluate the sorbent ability of the aerogel. These organic molecules are characterized by different molecular weights and different numbers of sulfonate groups in their chemical structure, as reported in Figure 5. Indigo Carmine (Figure 5A and Cibacron Brilliant Yellow (Figure 5B) dyes have been widely used in various industrial fields, such as the textile industry, even though they have been considered toxic for humans, pigs and rats. Due to their high toxicity, wastes that contain these compounds have to be treated to minimize or eliminate their toxic effects.

The kinetic sorption tests were performed as already described in our previous paper. Very briefly, the lyophilized hydrogels were immersed inside a vial containing the solutions of the dyes at desired concentration (100 mg/L and 350 mg/L) with a ratio between the mass of adsorbent material and the dye’s solution volume of 1.33 mg/mL.

At fixed time points, the sampling was performed, each time taking 1 mL of the solution, which was then put back again in the system after the analysis. The mass of the adsorbed dye per unit of mass of adsorbent material, defined sorption capacity *q*, and the percentage of dye removal, were evaluated at each time through Equations (1) and (2).

(1) $$q=\frac{m_{0}-m_{t}}{m_{HG}}$$

(2) $$\%\;dye\;removed=\frac{m_{0}-m_{t}}{m_{0}}$$

where *m_0_* and m_t_ are the masses in mg of the dye pollutant in the volume of solution at the beginning and at time t, respectively; and *m_HG_* is the mass in g of the lyophilized hydrogel. Then, isotherm tests were carried out with different concentrations for each dye using a ratio between the mass of the adsorbent material and the volume of the solution of 0.8 mg/mL and leaving the system in this condition for a long time (4 h) to reach equilibrium. The concentration range of 0–300 mg/L was selected to collect the data for the isotherm with CBY, and 0–150 mg/L was employed for IC. The data were modeled via non-linearized methods, and the Langmuir model was the one that best fitted the considered statistic. The Langmuir model, which assumes that the sorbent is coated by a monolayer of the adsorbate, correlates the sorption capacity of the sorbent material (*q_e_*), calculated as mg of pollutants adsorbed per g of sorbent material, with the concentration of the pollutant in the solution at the equilibrium (*C_e_*) as reported in Equation (3).

(3) $$q_{e}=\frac{Q_{max}KC_{e}}{1+KC_{e}}$$

*Q_max_* is maximum adsorption capacity (mg/g), and *K* is the Langmuir isotherm constant or affinity constant (dm^3^/mg).

### 4.5. Desorption and Reusability Test

Desorption and reusability tests were conducted to evaluate the possibility of reusing the aerogels. The used samples were filtered on Buchner and washed with alkaline solvent (NaOH, 0.1 N) to release the dye adsorbed from the material. Then, the aerogels were reactivated in an acidic medium (HCl, 0.01 N) to re-protonate the framework of the material, and finally, the reusability tests were conducted on the regenerated sponges, always using the same concentration of 100 mg/mL.

### 4.6. Spectroscopy Analysis

Quantitative spectrophotometric analysis allows one to quantify the concentrations of substances through the measurement of the absorption of UV-vis radiation by the molecules [48,49,50]. The solution that should be analyzed absorbs incident radiation with a selected wavelength equal to the characteristic λ_max_, obtained from the UV-vis spectrum of the considered dye, and a final detector measures the intensity of the radiation exiting the sample. As is well-known, the absorbance of a sample and its concentration are linearly correlated through the Lambert–Beer law, reported in Equation (4), which is valid only at low concentrations.

(4) A=ε C l

*l* is the optical path in cm negligible for the employed cuvette; *ε* is the molar extinction coefficient characteristic of each substance and represents the absorbance of the sample at a unitary concentration and a unitary optical path; and *C* is the molar concentration of the sample.

### 4.7. Statistical Analysis

Experimental data were analyzed using analysis of variance (ANOVA). Statistical significance was set to *p* value < 0.05. The results are presented as mean value ± standard deviation.

## Figures and Tables

**Table 1:** Parameters of the Langmuir isotherm model for CBY and IC.

Dye	Qmax [mg/g]	K [L/mg]
IC	457.67	0.11
CBY	748.78	0.07

**Table 2:** Performances obtained in this work compared with the literature.

Material	Dyes Adsorbed	Maximum Adsorption Capacity [mg/g]	Reference Paper
GO/CS AG	Indigo Carmine	457.7	This paper
Adsorbent material based corn stover and paper waste	Indigo Carmine	148.8	[36]
Mesoporous Mg/Fe layered double hydroxide nanoparticles	Indigo Carmine	62.5 (acid conditions)	[37]
Bi_2_O_3_ doped MGO	Indigo Carmine	126.6	[38]
GO/CS AG	Cibacron Brilliant Yellow	748.8	This paper
Functionalized chitosan beads	Cibacron Brilliant Yellow	179.5	[39]
Nanocarbons	Cibacron Brilliant Yellow	300	[40]
Activated commercial carbons	Cibacron Brilliant Yellow	527	[40]

## Data Availability

The data generated or analysed during this study are available from the corresponding author on reasonable request.

## Supplementary Materials

The following are available online at https://www.mdpi.com/article/10.3390/gels9020110/s1. Figure S1: SEM images of GO/CS AGs with the respective magnifications. Figure S2: Calibration curve for Indigo Carmine. Figure S3: Calibration curve for Cibacron Brilliant Yellow. Figure S4: ATR FT-IR spectrum of GO-CS aerogels [51]. Table S1: Numerical values of the parameters obtained through the nano-computed tomography.
