# Supplementary material for: On the Sorbent Ability and Reusability of Graphene-Oxide–Chitosan Aerogels for the Removal of Dyes from Wastewater
Source: Gels. 2023 Jan 27;9(2):110. doi: 10.3390/gels9020110 (PMC9956623; doi:10.3390/gels9020110)
Supplement: Supplementary file 1 [file gels-09-00110-s001.zip › gels-2172556-supplementary.pdf]

Supplementary Materials

On the Sorbent Ability and Reusability of Graphene Oxide – Chitosan Aerogels

Filippo Pinelli [a], Chiara Piras [a], Filippo Rossi [a,*]

[a] *Department of Chemistry, Materials and Chemical Engineering "Giulio Natta", Politecnico di Milano, via Mancinelli 7, 20131, Milan, Italy*

* Corresponding authors

E-mail address: filippo.rossi@polimi.it

1. SEM analyses of the GO/CS AGs

As already discussed in the main part of the paper, the GO/CS AGs have been characterized using SEM analysis. Here we report, in Figure S1, some additional images.

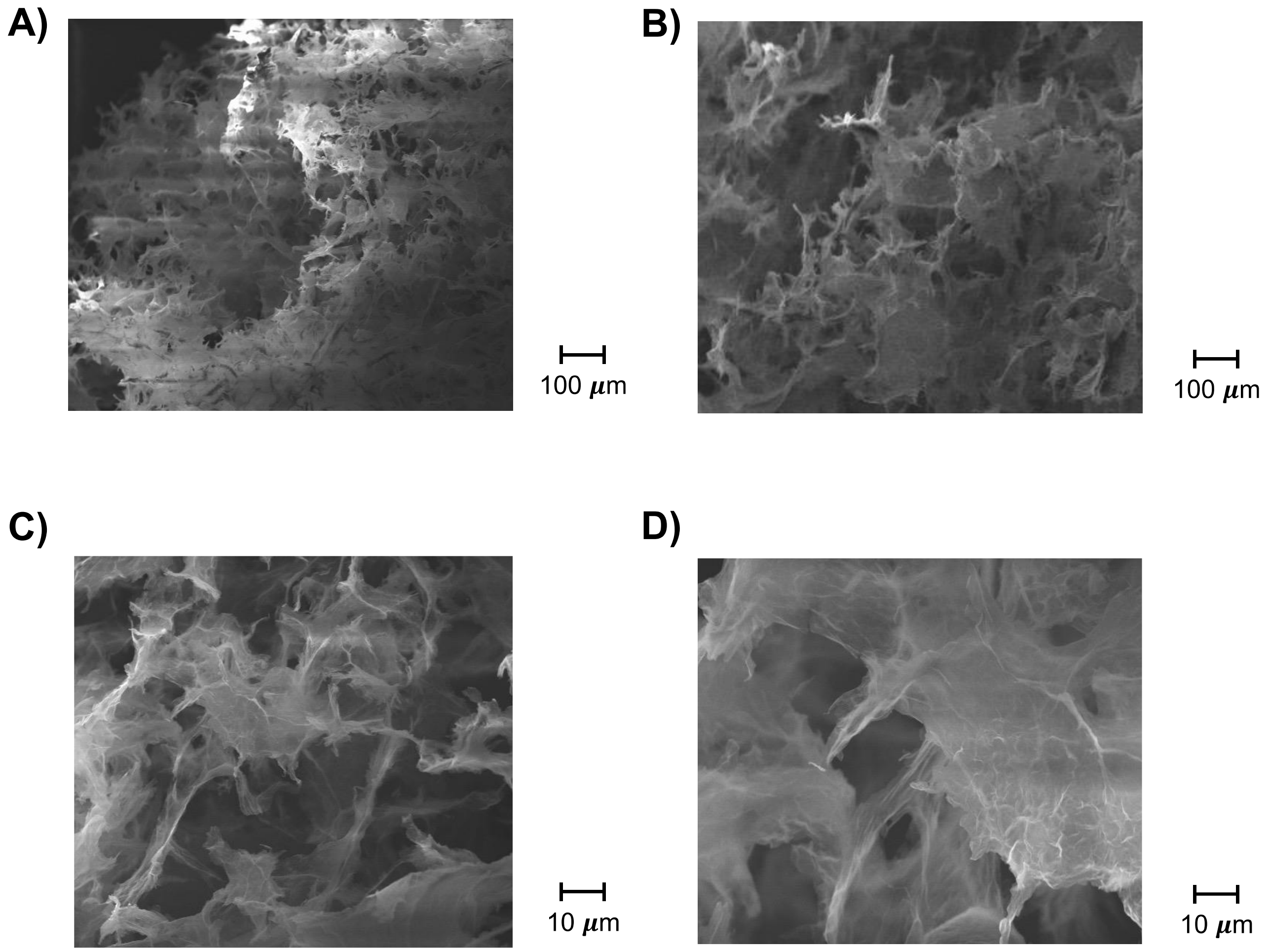

Figure S1 SEM images of GO/CS AGs with the respective magnifications.

2. Nano computed Tomography

In the following Table S1 additional parameters and information regarding the 3D-anaylisis performed through the nano-computed tomography on the GO/CS AGs are reported.

Dataset	Value
Object surface / volume ratio	1.026 μm^{-1}
Object surface density	0.032 μm^{-1}
Structure thickness	2.986 μm
Structure separation	71.61 μm
Structure linear density	0.011 μm^{-1}
Fractal dimension	2.169

Close porosity	0.02 %
Open porosity	96.862 %
Total porosity	96.88 %
Connectivity density	0.00003 μm^3
Standard dev. of structure thickness	1.157 μm
Standard dev. of structure separation	28.894 μm

Table S1. Numerical values of the parameters obtained through the nano-computed tomography.

3. Calibration curves for the dyes

Quantitative spectrophotometric analysis allowed to quantify the concentration of substances through the measurement of the absorption of UV-vis radiation by the molecules. The use of calibration curves was essential to apply the Lambert-Beer law. Here, in figure S.2, we report the calibration curve employed for indigo carmine and in figure S.3 the one for cibacron brilliant yellow.

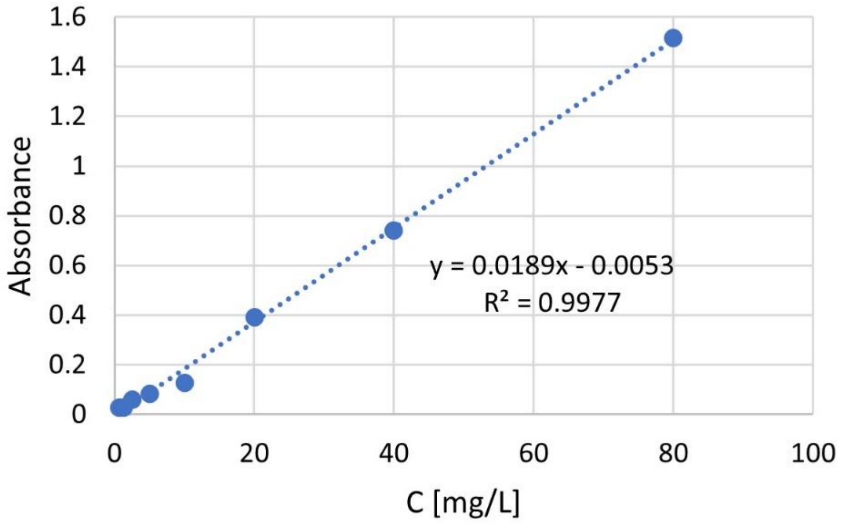

Figure S2. Calibration curve for indigo carmine.

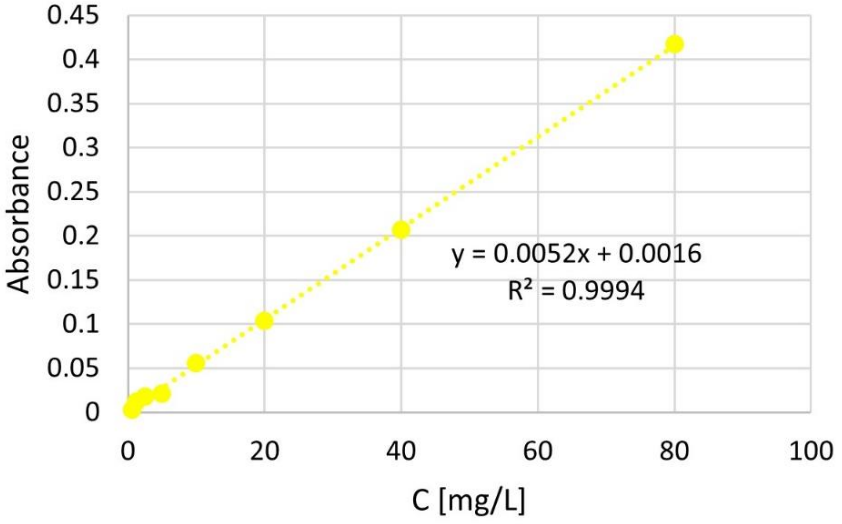

Figure S3. Calibration curve for cibacron brilliant yellow.

4. IR and NMR spectra

In the following figure S.4 we report the ATR-FTIR spectrum of the GO-CS aerogels. In accordance with previous work [23] and literature [42, 51] the IR spectrum of the aerogels produced shows characteristic bands centered at 1100 (**3**) and 900 (**4**) cm^{-1}, corresponding to signatures assigned to a glucopyranose ring unit, whereas the C=O stretching vibration of amide I (NHCO) and amide II (N−H) bending of NH_2 is observed at 1650 (**1**) and 1570 (**2**) cm^{-1}. The formation of an amide linkage between GO and chitosan can be demonstrated by IR intensity of the amide bands observed. This provides support for the formation of a linkage between GO and chitosan.

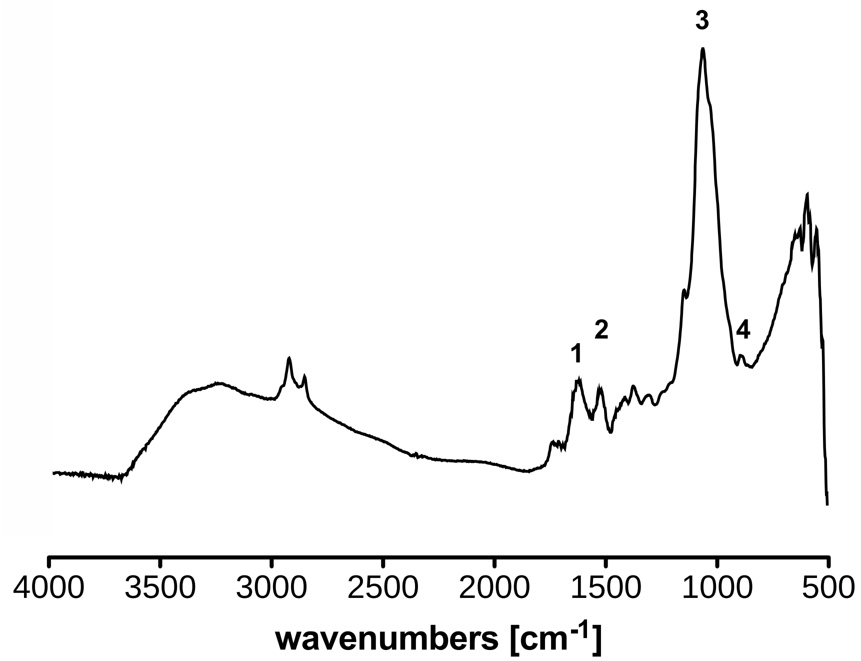

Figure S4. ATR-FTIR spectrum of the GO-CS aerogels.
